# No Evidence for Selection of HIV-1 with Enhanced Gag-Protease or Nef Function among Breakthrough Infections in the CAPRISA 004 Tenofovir Microbicide Trial

**DOI:** 10.1371/journal.pone.0071758

**Published:** 2013-08-28

**Authors:** Denis R. Chopera, Jaclyn K. Mann, Philip Mwimanzi, Saleha Omarjee, Xiaomei T. Kuang, Nonkululeko Ndabambi, Sarah Goodier, Eric Martin, Vivek Naranbhai, Salim Abdool Karim, Quarraisha Abdool Karim, Zabrina L. Brumme, Thumbi Ndung'u, Carolyn Williamson, Mark A. Brockman, the CAPRISA 004 TRAPS Team

**Affiliations:** 1 HIV Pathogenesis Programme, Doris Duke Medical Research Institute, Nelson R. Mandela School of Medicine, University of KwaZulu-Natal, Durban, South Africa; 2 KwaZulu-Natal Research Institute for Tuberculosis and HIV, Nelson R. Mandela School of Medicine, University of KwaZulu-Natal, Durban, South Africa; 3 Institute of Infectious Disease and Molecular Medicine, and the Division of Medical Virology, University of Cape Town and National Health Laboratory Services, Cape Town, South Africa; 4 Faculty of Health Sciences, Simon Fraser University, Burnaby, British Columbia, Canada; 5 British Columbia Centre for Excellence in HIV/AIDS, Vancouver, British Columbia, Canada; 6 Centre for the AIDS Programme of Research in South Africa, Doris Duke Medical Research Institute, Nelson R. Mandela School of Medicine, University of KwaZulu-Natal, Durban, South Africa; 7 Max Planck Institute for Infection Biology, Berlin, Germany; Lady Davis Institute for Medical Research, Canada

## Abstract

**Background:**

Use of antiretroviral-based microbicides for HIV-1 prophylaxis could introduce a transmission barrier that inadvertently facilitates the selection of fitter viral variants among incident infections. To investigate this, we assessed the in vitro function of *gag-protease* and *nef* sequences from participants who acquired HIV-1 during the CAPRISA 004 1% tenofovir microbicide gel trial.

**Methods and Results:**

We isolated the earliest available *gag-protease* and *nef* gene sequences from 83 individuals and examined their in vitro function using recombinant viral replication capacity assays and surface protein downregulation assays, respectively. No major phylogenetic clustering and no significant differences in *gag-protease* or *nef* function were observed in participants who received tenofovir gel versus placebo gel prophylaxis.

**Conclusion:**

Results indicate that the partial protective effects of 1% tenofovir gel use in the CAPRISA 004 trial were not offset by selection of transmitted/early HIV-1 variants with enhanced Gag-Protease or Nef fitness.

## Introduction

The placebo-controlled CAPRISA 004 trial demonstrated a 39% reduction in HIV-1 acquisition through vaginal use of a 1% tenofovir microbicide gel in women [1]. Previous studies of the Tenofovir gel Research for AIDS Prevention Science (TRAPS) cohort, which comprises all 98 incident HIV infections from CAPRISA 004, reported that tenofovir gel use was not associated with increased selection of antiretroviral resistance mutations [1] nor did it alter the number of transmitted/founder variants [2]. However, it remains important to ensure that antiretroviral-based strategies for HIV prophylaxis do not introduce sieving effects [3] or result in the selection of viral variants with enhanced fitness or pathogenesis in individuals with breakthrough infection. The functional characteristics of an individual's virus can influence the disease course [4], [5], [6], [7], [8], [9], [10], [11]; as such, inadvertent selection of fitter HIV-1 strains at or shortly after transmission may have clinical implications.

While there was no obvious influence of 1% tenofovir gel on the infecting virus based on *pol* and *env* sequencing in the TRAPS cohort [1], [2], it is important to rule out more subtle effects of microbicide use on transmitted viruses with other genetic or functional differences. Building on previous studies that demonstrated substantial inter-individual differences in *gag-protease* replication capacity [4], [10] and *nef* function [7], [9], we investigated whether tenofovir gel prophylaxis was associated with differences in the in vitro function of early/transmitted viral Gag-Protease and Nef proteins.

## Methods

### Ethics Statement

This study was approved by the Biomedical Research Ethics Committee at the University of KwaZulu-Natal (South Africa). All participants provided written informed consent for samples to be stored and used for this study. The clinical trials registration number of the parent trial (CAPRISA004) was NCT00441298.

### Study participants and Samples

The Tenofovir gel Research for AIDS Prevention Science (TRAPS) cohort comprises all 98 participants who acquired HIV-1 during the CAPRISA 004 microbicide trial [1]. We examined baseline plasma samples from 31 (of 38; 81.6%) 1% tenofovir gel and 52 (of 60; 86.7%) placebo gel recipients (84.7% of total infections) for whom the sample was collected within 6 months of the estimated date of infection (median [IQR] 37.0 [24.25–55.75] days post-infection). There was no difference in median time post-infection between samples from the treatment and the placebo arms of the trial (37.0 vs. 38.0 days, respectively, p = 0.3).

### Gag-protease and nef amplification and functional assessment

HIV-1 *gag-protease* and *nef* gene regions were amplified from plasma HIV RNA by nested RT-PCR using sequence-specific primers, and bidirectionally sequenced on an ABI 3730xl automated DNA sequencer (Applied Biosystems). All sequences except for one were confirmed as HIV-1 subtype C using the recombinant identification program (RIP; http://www.hiv.lanl.gov/content/sequence/RIP/RIP.html). A single individual from the placebo arm harbored a recombinant subtype A/D infection and was excluded from subsequent analyses, yielding 31 tenofovir and 51 placebo recipients for study. Alignment of sequences to the HIV-1 reference strain HXB2 was performed using an in-house tool based on the HyPhy platform [12]. Phylogenetic trees were constructed using maximum-likelihood approaches (PhyML; [13]) and visualized using FigTree (http://tree.bio.ed.ac.uk/software/figtree/).

Generation of quasispecies-containing recombinant viruses expressing patient-derived *gag-protease* sequences in an NL4-3 backbone (described in [4], [10]) was successful for 30 tenofovir (97%) and 45 placebo (88%) participants. In vitro replication capacity of Gag-Protease recombinant viruses was measured by flow cytometry using a GFP reporter T-cell assay, and results were normalized to the HIV-1 subtype B laboratory adapted reference strain NL4-3 as described previously [4], [10].

The *nef* region was successfully amplified from plasma HIV RNA for 31 tenofovir (100%) and 48 placebo (94%) participants. For each participant, a single *nef* sequence that clustered closely with the original bulk sequence by phylogenetic analysis was cloned into the pSELECT-GFPzeo vector (InvivoGen, Inc.). The ability of each Nef clone to downregulate surface CD4 and HLA-A*02 molecules was measured by flow cytometry following transfection of CEM T cells, as described previously [9], [14]. All results were normalized to a positive control Nef clone derived from HIV-1 subtype B strain SF2. In vitro expression of each Nef clone was verified in CEM T cell lysates by Western blotting using two independent primary Nef antisera [9], and results were normalized to actin and compared to expression of SF2 Nef control. Western blot band intensities were quantified using ImageQuant LAS 4000 (GE Healthcare Life Sciences). Gag-protease and Nef functional measurements were performed in duplicate in independent experiments and results are reported as the mean of these measurements.

Bulk *gag* and clonal *nef* nucleotide sequences have been deposited into GenBank, accession numbers KF208740 – KF208816 for *gag*, and KF208817 – KF208898 for *nef*.

### Statistical Analyses and power estimates

Wilcoxon rank-sum tests were used to compare Gag-Protease replication capacity, Nef CD4 downregulation and Nef HLA downregulation functions between the tenofovir gel and placebo arms. Spearman's correlation was used to test concordance between replicate measurements, and to investigate associations between viral protein functions and clinical markers of disease progression (plasma viral load and CD4+ cell counts). Statistical tests were implemented in Prism 5.0 (GraphPad Software, Inc.). Conservative power estimates based on a total sample size of 75 and published measures of central tendency from assessments of subtype C sequences using identical methods [10] indicate that the current study possesses >80% power to detect functional differences of 6.5% between trial arms at α = 0.05.

## Results

**Figure 1:**
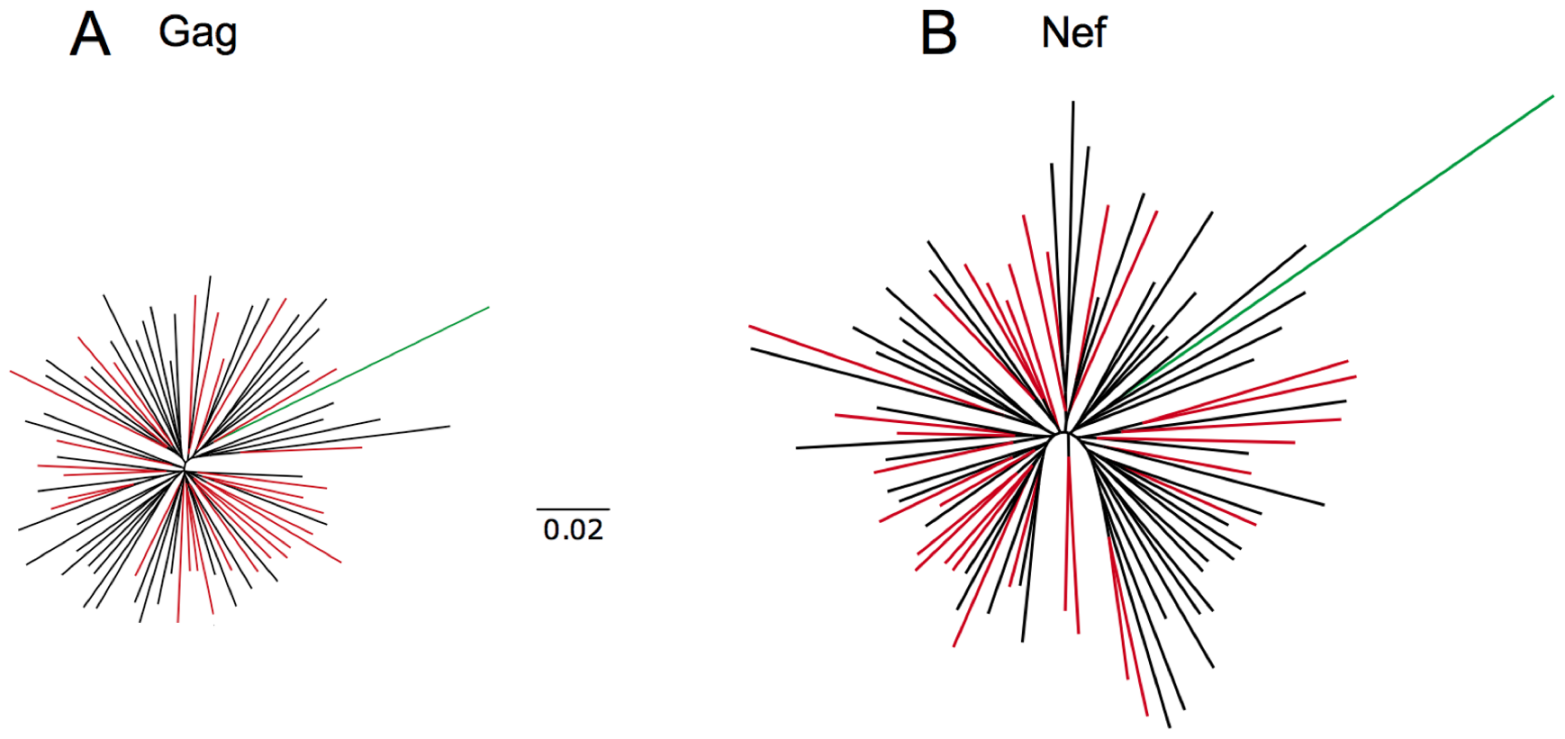
Sequence analyses of HIV-1 Gag and Nef from treatment and placebo arms. Maximum-likelihood phylogenetic trees of bulk *Gag* sequences (Panel A) and clonal *Nef* sequences (panel B) from participants who received 1% tenofovir gel (red) or placebo (black) demonstrated no major clustering between arms of the trial. HIV-1 subtype B reference sequence HXB2 is shown in green.

To examine whether the use of 1% tenofovir microbicide gel selected for transmitted/early HIV-1 strains with enhanced fitness, we assessed the sequence and in vitro function of plasma RNA-derived *gag-protease* and *nef* genes from the majority of incident HIV-1 infections in the CAPRISA 004 trial. Bulk *gag* and clonal *nef* sequences from tenofovir and placebo gel participants did not display substantial phylogenetic segregation (Figure 1), ruling out major lineage effects of viral species that infected the two groups.

**Figure 2:**
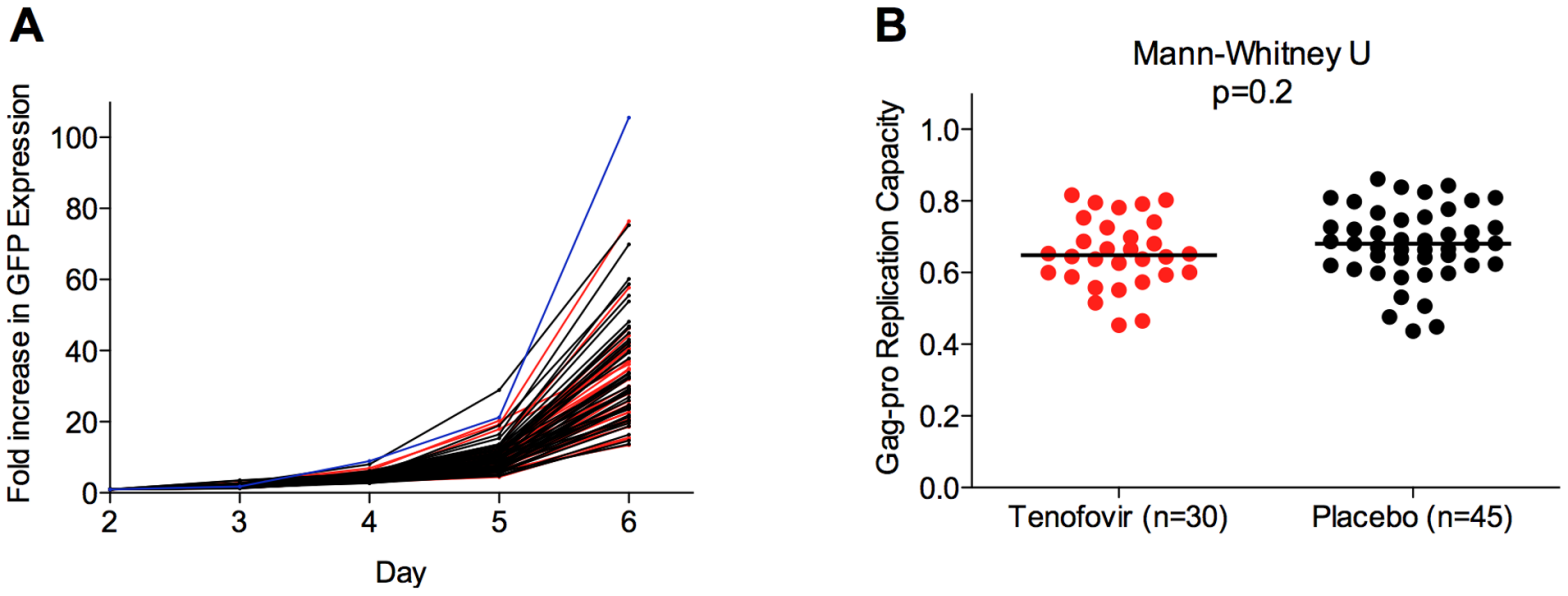
Replication capacity of recombinant viruses expressing *Gag-Protease* sequences from 1% tenofovir gel and placebo participants. Panel A: Replication data are shown for recombinant NL4-3 viruses encoding *gag-protease* from participants in the 1% tenofovir gel (red) and placebo (black) study arms, expressed as fold-increase in GFP expression over 6 days, relative to day 2. WT NL4-3 control is indicated in blue. Panel B: The replication capacities of recombinant viruses encoding participant-derived *gag-protease* sequences, calculated as the slope of viral spread normalised to WT NL4-3, are shown. No signifant difference in Gag-Protease function was observed between study arms (Mann-Whitney, p = 0.2).

Gag-Protease replication capacity was measured for 75 samples using a recombinant virus GFP reporter T cell assay [4] (Figure 2). Duplicate measurements of recombinant viral replication capacity were highly concordant (Spearman's correlation, r = 0.84 and p<0.0001, not shown). Replication capacity was calculated as the slope of increase in the percentage of HIV-infected (GFP+) cells from days 3–6 post-infection and normalized to that of the HIV-1 subtype B reference strain NL4-3, such that a value of 1.0 indicates a replication capacity equal to that of NL4-3, whereas values of >1.0 or <1.0 indicate replication capacities higher or lower than NL4-3, respectively. The replication capacity value of a given recombinant virus was not influenced by minor differences in the percentage of infected cells on day 3 (not shown). We observed no significant difference between the replication capacities of recombinant viruses encoding Gag-Protease sequences between individuals who received 1% tenofovir gel (n = 30) versus placebo (n = 45) (median [IQR] 0.65 [0.59–0.73] and 0.68 [0.62–0.75], respectively, Wilcoxon rank-sum test, p = 0.2) (Figure 2).

**Figure 3:**
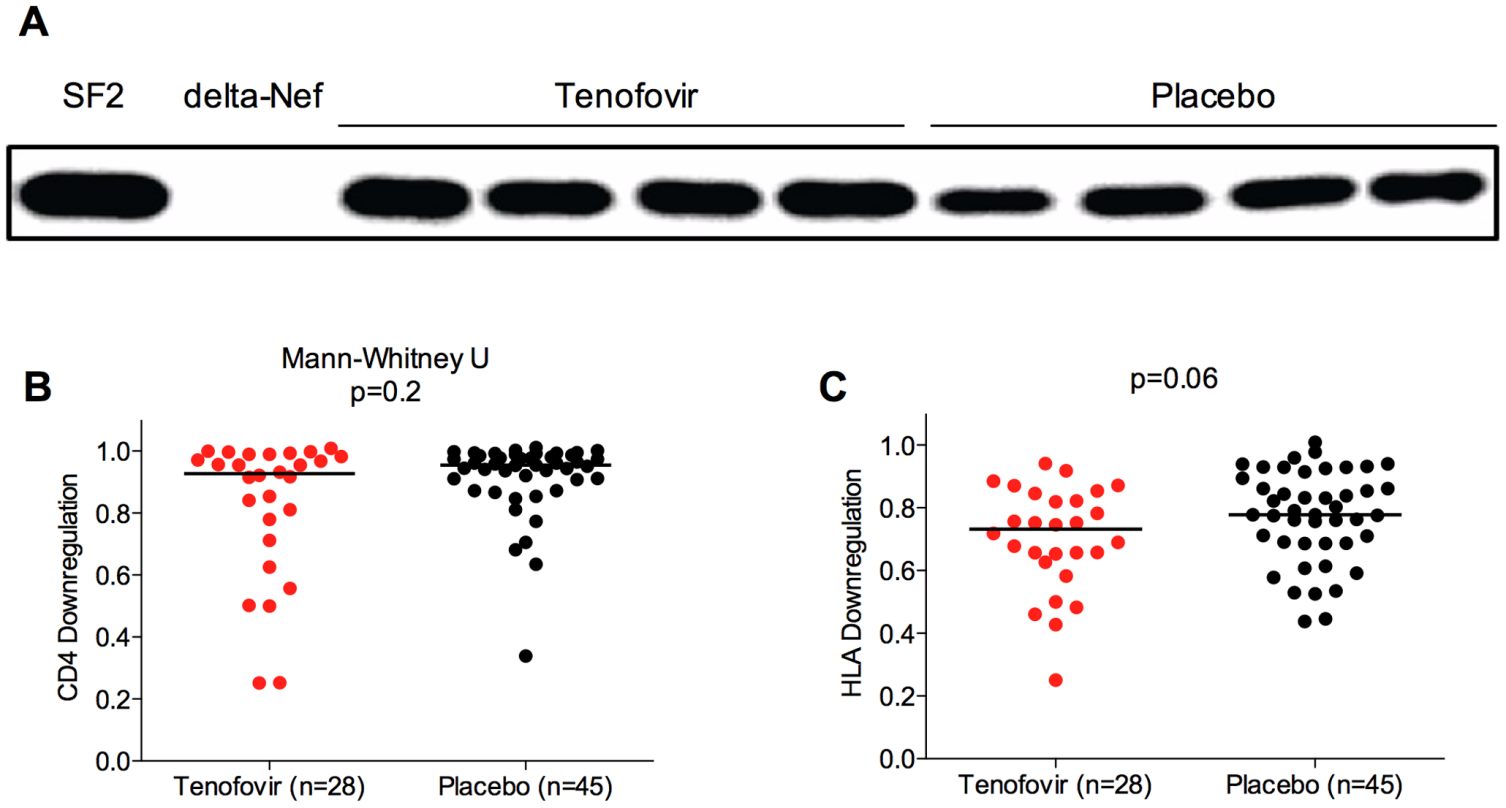
Functional analyses of Nef clones from treatment and placebo arms. Panel A: Selected Western blot results depict control SF2 Nef, empty (delta-Nef) plasmid and four samples each obtained from participants in the 1% tenofovir gel and placebo study arms. Panel B: Results for the CD4 downregulation activity of participant-derived *nef* isolates are shown, normalized to control SF2 Nef (which is equal to 1.0). Panel C: Results for the HLA-A*02 downregulation activity of participant-derived *nef* isolates are shown, normalized to control SF2 Nef (equal to 1.0). No significant differences in Nef-mediated CD4 or HLA downregulation function were observed between study arms (Mann-Whitney, p = 0.2 and p = 0.06, respectively).

We next assessed the ability of 79 participant-derived Nef clones to downregulate surface CD4 and HLA class I molecules using a transient CEM T cell transfection assay [9]. To verify expression of the plasma-derived *nef* genes following DNA transfection, proteins were detected by Western blot using two anti-Nef primary antibodies and results normalized to cellular actin and compared to control SF2 Nef (Figure 3A). Of the Nef clones assessed, six (8%) displayed low expression (<0.25 of SF2 control) as well as poor CD4 and HLA downregulation functions (<0.4 of SF2 control). Although it is possible that these six clones represented authentic *nef* sequences that would display similar in vivo defects, we excluded these clones from further analysis because poor in vitro expression, stability, and function resulting from PCR or cloning errors could not be ruled out.

For the remaining 73 Nef clones whose steady-state protein expression levels were comparable by Western blot, we examined CD4 and HLA downregulation function and normalized results to that of control SF2 Nef. As such, a value of 1.0 indicates an ability to downregulate the relevant cell-surface molecule (CD4 or HLA) to a level comparable to that of the control, whereas values of >1.0 or <1.0 indicate downregulation activities higher or lower than that of SF2, respectively. We observed excellent correlation between duplicate measurements (Spearman's r = 0.85; p<0.0001 and r = 0.86, p<0.0001 for CD4 and HLA downregulation, respectively). Nef clones from tenofovir gel (N = 28) and placebo (N = 45) participants displayed no significant difference in Western blot band intensity compared to actin-normalized control SF2 Nef (median [IQR] 0.76 [0.64–0.86] and 0.79 [0.63–0.92], respectively, p = 0.5). Similarly, no relationship was observed between Nef Western blot band intensity and CD4 (r = 0.03, p = 0.8) or HLA (r = 0.1, p = 0.3) downregulation functions. CD4 downregulation activity was comparable between Nef clones obtained from 1% tenofovir gel and placebo participants (median [IQR] 0.93 [0.73–0.99] and 0.96 [0.89–0.99], respectively, p = 0.2) (Figure 3B). However, we observed a trend towards lower HLA class I downregulation function in Nef clones from tenofovir gel participants compared to those who received placebo (median [IQR] 0.73 [0.63–0.84] and 0.77 [0.69–0.90], respectively, p = 0.06) (Figure 3C). We conducted a sensitivity analysis on the entire Nef dataset (including the six samples with low expression/function), which yielded similar results (p = 0.2 and p = 0.06 for CD4 and HLA downregulation, respectively, not shown). Overall, when the function of all Nef sequences was assessed together, we observed a significant correlation between Nef-mediated CD4 and HLA downregulation activities (Spearman's r = 0.3, p = 0.007, not shown).

**Figure 4:**
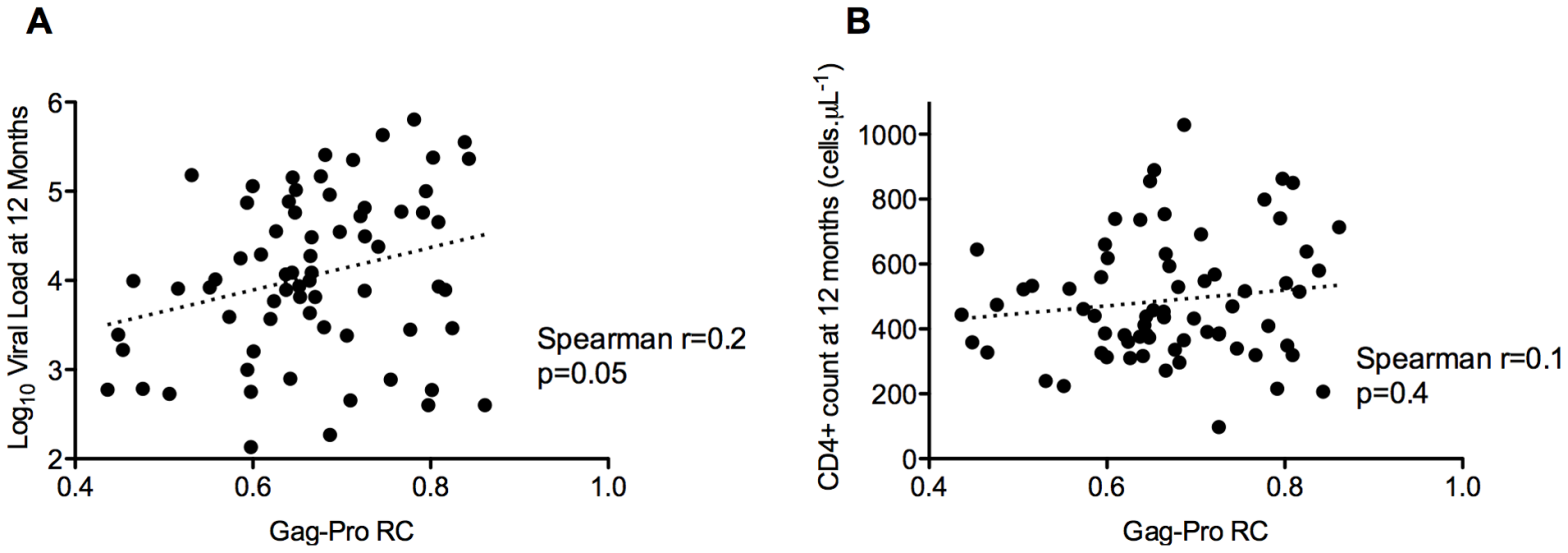
Association between baseline Gag-Protease replication capacity and HIV-1 clinical parameters at 12-months post-infection. A modest correlation was observed between early Gag-Protease-mediated replication capacity and plasma viral load (Spearman, r = 0.2, p = 0.05; Panel A), but not CD4+ cell count (Spearman, r = 0.1, p = 0.4; Panel B), at 12 months post-infection.

Finally, we evaluated potential relationships between *gag-protease* and *nef* gene functions and the clinical status of study participants. No correlations were observed between Gag-Protease replication capacity and the ability of Nef to downregulate either CD4 or HLA when data from the trial arms were analyzed separately or together (not shown). Among the 76 participants for whom HIV-1 clinical measurements were available at 12 months post-infection, a modest yet statistically significant correlation was observed between baseline Gag-Protease replication capacity and viral load at 12 months (Spearman's r = 0.2, p = 0.05, Figure 4A), but no such association was observed with CD4+ cell counts at 12 months post-infection (Figure 4B). Furthermore, no relationship was observed between baseline Nef-mediated CD4 or HLA downregulation function and plasma viral load (Spearman's r = 0.05, p = 0.7 and r = 0.1, p = 0.4, respectively, not shown), or CD4 count (Spearman's r = 0.1, p = 0.3; and r = −0.09, p = 0.5, respectively, not shown) at 12 months post-infection.

## Discussion

To examine the possibility that transmitted/early HIV-1 strains with altered function could be inadvertently selected by antiretroviral-based microbicide prophylaxis, we assessed plasma RNA *gag-protease* and *nef* sequence and function for the earliest available time points (median 5 weeks following estimated date of infection) from participants who acquired infection during the CAPRISA 004 1% tenofovir gel microbicide trial [1]. We observed no difference in the in vitro replication capacity of recombinant viruses expressing Gag-Protease quasi-species from individuals who received tenofovir gel versus placebo. Similarly, the ability of representative participant-derived Nef clones to downregulate cell-surface CD4 or HLA class I was not significantly different between study arms, although Nef clones from participants who received tenofovir gel exhibited HLA downregulation activities that were on average 3% lower than those from participants in the placebo arm (p = 0.06). At present, the biological implications of this observation remain unclear. Of interest, an earlier study on this cohort reported that participants in the tenofovir gel arm displayed higher Gag-specific CD4+ T cell responses [15], raising the intriguing possibility that reduced Nef function could contribute to differences in antigen presentation among trial participants.

The modest yet statistically significant correlation between in vitro Gag-Protease replication capacity and plasma viral load at 12 months in the overall cohort is consistent with previous reports [4], [10] and supports the contribution of *gag-protease* during HIV-1 pathogenesis. Other groups have reported conflicting associations between Nef-mediated CD4 or HLA downregulation function and clinical markers of disease [7], [9], [16], [17]; however, we observed no association between these factors in our study.

In summary, our analysis of baseline samples from the majority of incident HIV-1 cases in the CAPRISA 004 trial indicates that the use of 1% tenofovir microbicide gel was not associated with significant alterations in Gag-Protease or Nef function in vitro. The partial protective effects of tenofovir observed in this trial are therefore unlikely to be offset by inadvertent selection of transmitted/early HIV-1 variants with enhanced viral fitness.
